# The Recent Investigations of Ricord and Others upon the Subject of Syphilis

**Published:** 1865-03

**Authors:** E. Andrews

**Affiliations:** Prof. of Surgery in Chicago Medical College


ARTICLE VIII.

### THE RECENT INVESTIGATIONS OF RICORD AND OTHERS UPON THE SUBJECT OF SYPHILIS.

By E. ANDREWS, M.D., Prof. of Surg. in Chic. Med. Col.

One of the most positive achievments in surgical pathology, consists in the rediscovery of the double nature of syphilitic poison.

It is satisfactorily proven by the investigation of RICORD and others, that the disease variously called soft chancre, chancroid, chancrelle, non-infecting chancre, &c., never contaminates the constitution, and is essentially different from the true infecting chancre of syphilis. The reasons of this conclusion I will briefly recapitulate.

First—as to the history of these diseases. It is a fact demonstrable, that while the non-infecting chancre, or chancroid, has been known from all antiquity, and is described and referred to by Hippocrates, Celsus, and others, there is nowhere a record or a trace of genuine syphilis previous to the time of its outbreak in Europe, just before the year 1500. For instance, the ancient writers rank the occurrence of painful suppurating buboes among the prominent symptoms of the venereal disease known to them, while in genuine syphilis, buboes are seldom prominent enough to attract much attention, and very rarely suppurate. The inflammatory suppurating bubo is a characteristic mark of the chancroid only. I have discovered in ancient history a curious little passage, which I have not seen referred to by any author, and which shows that troublesome inflammatory buboes were well known to the Egyptians, and supposed by them to date back beyond the days when the Israelites left that country under the lead of Moses.

The historian Josephus wrote two controversial treatises defending the Jews against the slanders of Apion, an Egyptian author. In the Second Book, he indignantly quotes Apion as giving the following account of the origin of the Jewish Sabbath, viz.: that after the Israelites left Egypt, they travelled six days towards Palestine, when they were attacked with buboes in their groins, on account of which they were compelled to stop and rest on the seventh day, being unable to march. Therefore, as the Egyptian name of a bubo was *Sabbo*, they called it the *Sabbath-day*, that is, *Bubo-day.* Josephus combats this etymology by saying that it is true that *Sabbo* in Egyptian signifies bubo, but that *Sabbath* is a Hebrew word, and means not *bubo*, but *rest.*

Hippocrates and Celsus were well acquainted with chancroids and suppurating buboes, but give no account of genuine syphilis. The writings of Solomon have been supposed to prove the existence of constitutional syphilis, because they plainly refer to some disease as causing the death of such men as resort to the houses of strange women. Solomon gives no description of this disease, but the tone of his language conveys the impression of some quick and rapid attack which brought the offender promptly to his grave. This corresponds to the sloughing form of chancroid which is especially prevalent in hot climates, and which often destroys life in a few days. I have seen two fatal cases of the same sort in this city. Genuine syphilis, on the contrary, is slow in developing, and wastes away the patient through years of suffering.

All ancient descriptions and referenees, therefore, to venereal sores, seem to point to the chancroid only. This continued to be the case down to the year 1494, when about the time of the famous siege of Naples by the French, genuine syphilis made its appearance. It is a matter of history that the physicians of that day never confounded these two diseases together, but distinctly recognised their separate character. In their books the two are found treated of in separate chapters, the old disease, or chancroid, with which they had long been familiar, being clearly described, and often called Caries, *non Gallica*, to distinguish it from the genuine *Gallica* or French disease. They attribute no constitutional to the former, but give full prominence to the evidences of general infection of the system by the latter.

The fact that the medical world at that time, though long familiar with chancroid, never confounded the new disease with the old, is powerful evidence of the aspect presented by these two diseases to unprejudiced minds. But as the old generation of physicians died out, who had seen chancroid for years in an unmixed form, their successors gradually became confused by the constant occurrence of the two diseases together. Hence it happened that soon after 1520, the chancroid and true syphilis began to be looked upon as the product of the same virus, and continued to be thus described down to our own day.*

*Diagnosis of these Diseases.*—Much confusion exists in the minds of the profession on this subject, and some of our most eminent authors commit egregious blunders in their remarks upon it.

Chancroid, sometimes called soft chancre, has no distinct period of incubation, but commences at once to produce its effects, and in forty-eight or seventy-two hours is usually large enough to show its character. It consists, in its normal form, of a rounded ulcer with a flat base, which is sunk into the mucous membrane with distinct edges, as though a piece of membrane had been cut out with a rough punch. The floor of the ulcer is not red, but is covered with a whitish diphtheritic exudation, and bathed in pus. The tissues beneath the ulcer preserve all their normal softness, which is a very important point of distinction between this and true syphilis, or hard chancre. It is proper to remark, however, that the base of a chancroid may be made to assume a fictitious hardness by irritating applications, such as nit. of silver, &c. The chancroid has but little tendency to spontaneous healing, and if not treated, will often continue a year or more in the same patient. The pus of this ulcer is capable of reproducing similar ulcers to an indefinite extent by inoculation, either in the same, or in other patients. These inoculations have been practised to ah enormous extent, and the results of careful and extensive experiments show that the pure chancroid never produces the symptoms of secondary syphilis. The buboes which follow chancroid commonly involve

* See Bumstead on Venereal Diseases.

only one or two glands, and are very prone to inflame or suppurate, showing in that respect the same aplastic and suppurative character as the chancroid from which they sprang. Finally, the chancroid in aplastic constitutions is liable to assume a plagedenic, or even a gangrenous form, and sometimes causes the death of the patient within a few days, with symptoms of prostration and pyæmia.

On the other hand, genuine syphilis, in its pure form, presents the following symptoms: First, there is a period of incubation, which is seldom less than eight or ten days. The chancre, when formed, is smaller in size, and much less formidable in appearance, than the chancroid. The base is but little excavated, the floor is of a natural rosy color, without any diphtheritic exudation, and does not produce a particle of pus, unless the surface has been subjected to some irritating influence. The base and circumference of the ulcer are infiltrated with plastic lymph, so as to give a sensation of hardness to the touch. Sometimes, however, the induration merely exists in a very thin stratum, like parchment, under the ulcer, which can be felt by pinching it edgewise between the thumb and finger. In cases where the patient has been the subject of true syphilis previously, this symptom is often modified to such an extent that no induration whatever can be perceived, the chancre having the appearance of a mere abrasion of the mucous membrane. True chancre often heals spontaneously in a short time, leaving only the secondary symptoms to mark the progress of the disease. Its secretions when inoculated into the same patient are utterly inert, but will reproduce a true chancre on any other person who has not had the disease. Of those who have once had syphilis, only a small proportion—probably about one-fourth—are capable of having it a second time. The inoculated virus of a pure chancre never produces chancroid, nor does the virus of pure chancroid ever result in chancre. The buboes of chancre are usually numerous, show but little tenderness or inflammation, and very rarely suppurate. They manifest the same indisposition to produce pus which exists in the chancre from which they sprung. Finally, chancre seldom or never assumes the phagedenic form; but in probably every instance it more or less infects the constitution, and, unless treated, its usual course is to produce, one after another, the symptoms classed as secondary and tertiary syphilis. It has been proved that blood, and also other fluids drawn from a patient laboring under the so-called secondary syphilis, even at a distance from the chancre, is capable of producing syphilis by incubation; a point of extreme importance as bearing on the proper selection of wet nurses and vaccine scabs. It also suggests caution to surgeons operating on contaminated patients.

Placing these two sets of symptoms in parallel columns, we see the ground of diagnosis very clearly:—

| CHANCROID. | SYPHILIS. |
|---|---|
| Has no period of incubation. | Has a period of incubation. |
| Is frequently multiple. | Chancre seldom multiple. |
| Color whitish yellow. | Color rosy. |
| Not indurated at base. | Indurated at base. |
| Produces pus abundantly. | Produces scarcely any pus. |
| Produces large single suppurating bubo. | Produces several small non-suppurating buboes. |
| Never contaminates the system. | Always affects the system. |
| Can be inoculated successfully into the same patient. | Cannot be inoculated successfully into the same patient. |
| May occur any number of times in the same person. | Generally can occur only once in the same person. |

*Mixed Chancre.*—Chancroid is the most common, by far, of these diseases; while a pure syphilis, unmixed with chancre, is, in Chicago at least, quite rare. Next after the pure chancroids, the most common form is a mixture or complication of the two diseases, called "Mixed Chancre." This is the genuine old "Hunterian Chancre" of the authors. It manifests the characteristics of both diseases. The ulcer has the purulent effusion and diphtheritic surface of chancroid, with the hardened base of true syphilis. It is slow of healing, like the former, and infects the constitution, like the latter.

*Practical Conclusions.*—The most obvious inference from these facts is, that insomuch as a pure chancroid does not affect the constitution, it does not require constitutional treatment. If its purity is undoubted, it is all-sufficient to treat a chancroid by from two to four cauterizations with fuming nitric acid, nitrate of mercury, or sulphuric acid. It will then commonly heal without difficulty.

Practically, however, the case is not thus simple. What appears during the first few days to be a chancroid, may be really a mixed disease, in which the induration has not yet been developed, because the time of incubation of the syphilitic part of the symptoms has not fully elapsed. Again, in a person who has had syphilis before, if he proves able to take it a second time, the induration may be entirely absent. For these reasons we are rarely able to say that a chancroid is not mixed with syphilis, whatever may be its present appearance, unless it has been in existence a long time. Disastrous results in the spread of syphilis have occurred in families in this city, in consequence of surgeons relying on the softness of a chancre, and deciding, therefore, that there was no danger of constitutional infection. My practice, therefore, is to lean to the safe side, and treat nearly all venereal ulcers as if they contained syphilitic virus, without waiting for a full development of the disease. There is no doubt of the fact that the absorption of the virus into the system is so rapid that, in true syphilis, the constitution has already received the influence before the induration of the chancre is developed. Hence it is in vain to rely on early cautery as a means of preventing secondary symptoms. The cautery, however, is necessary for the destruction of the chancroidal virus and the healing of the ulcer.

A point of great practical importance has been established by experiments upon the inoculability of secondary syphilis. It appears that the blood and other fluids drawn from any part of the body, where there is a syphilitic tubercle or other form of secondary disease, will produce a pure chancre, followed by constitntional disease. Hence vaccination, nursing, surgical operations, dissections, &c., are all capable of transferring the disease from one person to another.

A single word about remedies. It is now pretty generally admitted that, in primary and secondary syphilis, mercurials are far superior to all other medicaments. BUMSTED, however, is of the opinion that in proportion to the increase of time which has elapsed, mercurials become less and less useful, and iodide of potassium more potent. My practice is to gently mercurialize the patient first, and direct iodide of potassium to be taken for six months afterwards, in doses of five grains, four times a-day.

